# Perceptions of healthcare access among Lithuanians aged 65 and over during the COVID-19 pandemic

**DOI:** 10.3389/fpubh.2025.1504049

**Published:** 2025-03-04

**Authors:** Vytenis Kalibatas, Snieguole Kaseliene, Ramune Kalediene, Olga Mesceriakova, Skirmante Sauliune

**Affiliations:** Department of Health Management, Lithuanian University of Health Sciences, Kaunas, Lithuania

**Keywords:** COVID-19, older adults, healthcare services accessibility, perception, assessment of healthcare needs, Lithuania

## Abstract

**Aim:**

This study investigates the perceived accessibility of healthcare services among older adults in Lithuania during the COVID-19 pandemic. The study is significant as it sheds light on geographical, organizational, and financial healthcare access issues encountered by the older population.

**Methods:**

Conducted in January 2024, the study involved an anonymous questionnaire survey of 1,503 Lithuanian residents aged 65 and older.

**Results:**

The most frequently utilized healthcare services were consultations with a general practitioner (75.4%) 22.0% of respondents reported not receiving any healthcare services. 53.5% respondents were satisfied with travel time to specialists. Common challenges included difficulties in getting appointments with specialists (53.9%) and dentists (36.2%). Financial barriers led to unmet healthcare needs: 12.6% of the respondents did not receive needed services, 12.8% did not undergo recommended tests, and 14.2% did not purchase prescribed medications. Healthcare services were less accessible to elders with lower education, lower incomes, and those who self-rated health poorly (*p* < 0.05).

**Conclusion:**

Most respondents received the healthcare they needed during the pandemic and rated geographical access positively. However, some problems in organizational and financial access were disclosed. The observed social gradient indicates that socioeconomic factors significantly influence healthcare access, potentially increasing vulnerability among certain groups.

## 1 Introduction

The COVID-19 pandemic has profoundly impacted healthcare systems worldwide, disrupting routine healthcare services and exacerbating existing inequalities in healthcare access (1). Among the most affected populations were older adults, particularly those aged 65 and over, who were at the higher risk for severe outcomes from COVID-19, necessitating consistent and accessible healthcare services to manage both pandemic-related and routine health needs (2).

Older adults were disproportionately vulnerable during the COVID-19 pandemic due to a combination of biological, social, and health-related factors. Aging is associated with a natural decline in immune system function, reducing its ability to respond effectively to infections (3). Specifically, older adults exhibit diminished T-cell function and dysregulated inflammatory responses (4), both of which increase susceptibility to severe COVID-19 outcomes. SARS-CoV-2, the virus responsible for COVID-19, enters human cells via the ACE2 receptor. In older adults, ACE2 expression is often upregulated in certain tissues, potentially facilitating viral entry (5). Additionally, older individuals are more likely to have multiple comorbidities, further elevating their health risks. Chronic conditions such as hypertension, diabetes, cardiovascular disease, and chronic respiratory illnesses are strongly associated with severe COVID-19 outcomes and increased mortality. These comorbidities intensify the inflammatory response induced by SARS-CoV-2, leading to complications such as acute respiratory distress syndrome (6, 7). Age-related physiological changes also contribute to the heightened vulnerability of older adults. Reduced lung elasticity and decreased chest wall compliance diminish respiratory system reserve capacity, making it more difficult to manage respiratory infections (8). Furthermore, aging is associated with coagulation dysregulation, which increases the risk of thrombotic events—a condition worsened by COVID-19 (9).

Socioeconomic and behavioral factors also played a critical role in increasing the vulnerability of older adults during the pandemic. Social isolation and reduced access to healthcare resources due to lockdown measures disproportionately affected older populations (10). Moreover, heightened dependence on caregivers and residence in long-term care facilities placed many older individuals at elevated risk, as these congregate settings experienced widespread COVID-19 outbreaks (11). Mental health challenges, including anxiety and depression, were particularly pronounced among older adults, driven by fear of severe illness, social isolation, and experiences of bereavement (12).

Numerous studies have identified psychological, physical, and economic barriers that impact healthcare access for older adults, and these barriers were exacerbated during the COVID-19 pandemic, further contributing to disparities in healthcare availability (13, 14). The pandemic placed severe and far-reaching pressures on the acute healthcare system, significantly affecting older adults, who rely more heavily on acute medical services than younger populations (15). Restrictions on healthcare delivery led to a substantial rise in deferred routine care, including preventive visits, elective procedures, and non-COVID-19 hospital admissions (16).

In response to these challenges, telemedicine use surged as a critical alternative, enabling patients to access healthcare remotely and minimize exposure risks. Countries that reduced barriers to virtual care through telehealth reimbursement policies saw widespread adoption of these platforms. However, the increased reliance on telemedicine also underscored a digital divide: many older adults lack access to or familiarity with the necessary technologies, limiting their ability to benefit fully from remote healthcare services (17, 18).

Ensuring access to healthcare is critical to prevent illnesses and deaths from COVID-19 and non-COVID-19 cases in health systems that have deteriorated during the pandemic (19).Healthcare access is a multifaceted issue encompassing the availability, affordability, and acceptability of services. Perceived accessibility to healthcare refers to individuals’ assessments of the ease with which they can obtain necessary medical services. This concept is influenced by multiple factors, including geographical proximity to healthcare facilities, organizational efficiency in scheduling and providing care, and the financial burden of healthcare costs (20). The pandemic has significantly disrupted these aspects, with older adults facing increased difficulties in accessing care due to lockdown measures, the prioritization of COVID-19 cases, and personal fears of contracting the virus (21, 22). These disruptions could lead to delayed diagnoses, untreated chronic conditions, and overall deterioration in health status among older adults (23, 24).

The COVID-19 pandemic created significant barriers to healthcare access for patients with chronic diseases, particularly older adults. These challenges included service disruptions, facility closures, appointment cancelations, fear of infection, travel restrictions, and income loss. Together, these factors contributed to subjective unmet healthcare needs (25). Subjective unmet needs, defined as an individual’s perception of whether they received necessary care, serve as a critical measure of healthcare access barriers (26).

One clear impact of the pandemic and its accompanying restrictions was reduced access to healthcare services among older adults (27). In addition to feeling insecure and fearful of infection, many older individuals faced systemic service delays and disruptions to routine care. Several factors contributed to unmet healthcare demand during the pandemic: patients renounced medical care, care was postponed, or healthcare providers were unable to deliver services.

From a broader perspective, both supply- and demand-side factors influenced unmet needs. On the supply side, healthcare systems faced resource constraints, including a shortage of healthcare professionals, insufficient physical space, and an overwhelmed capacity focused on COVID-19 patients. On the demand side, factors such as fear of exposure, lack of transportation, adherence to lockdown measures, limited household budgets, and self-assessed perceptions of medical need discouraged care-seeking behaviors (28). Understanding the determinants of subjective unmet needs during the pandemic is crucial for future policy planning, as unmet healthcare needs can significantly affect health outcomes.

Socioeconomic factors played a significant role in healthcare access among older adults. Studies have shown that those with lower income, lower educational attainment, and those living in rural areas are more likely to encounter barriers to healthcare services (29). The pandemic has intensified these disparities, highlighting the need for targeted interventions to support the healthcare needs of disadvantaged older populations. While evidence of income-related horizontal inequities in unmet needs among European older adults was limited during the early waves of the pandemic, some countries must carefully monitor ongoing barriers to healthcare access. Delays in diagnosis and treatment could lead to adverse health outcomes, diminished quality of life, and potentially widen socioeconomic health inequalities among older populations (30).

To date, there is growing evidence on issues of access and utilization of health services for older people during the pandemic. Compared to other groups, older individuals face greater barriers to healthcare access and utilization due to factors including physical health limitations, disabilities, and mental health challenges. The findings of this study indicate that provider-level factors significantly influenced healthcare access for older adults during the pandemic. These factors encompassed both services related to COVID-19 diagnosis and treatment, as well as routine, non-COVID-19 care.

In addition to provider-level influences, various personal-level micro factors affect healthcare utilization among older adults. These include challenges with accommodation, attitudes toward aging, and levels of literacy and education. At a broader level, macro determinants, such as health policies, healthcare system structure, and an intricate combination of demographic, physical, social, cultural, and economic factors, also play a pivotal role in shaping access and utilization. Considering these factors comprehensively can provide policymakers with a holistic understanding, enabling the development of more equitable and effective interventions to improve healthcare access for older adults during the COVID-19 pandemic (31).

Investigating the perceived accessibility of healthcare services offers valuable insights into patient experiences and identifies barriers that objective measures may miss. Understanding perceived accessibility can reveal barriers related to both the health system (e.g., wait times, availability of services, financial constraints) and individual factors (e.g., socio-cultural aspects, technological barriers, personal health beliefs) (32). Given the increasing recognition of the importance of social determinants in influencing care for older adults (33), examining access and utilization of essential healthcare services during the pandemic remains crucial for informing future health policies and improving outcomes.

Scientific literature identifies multiple dimensions for measuring accessibility in healthcare. Previous studies suggested the notion of resistance of health systems and utilization power of populations to explain access from a broad perspective. Resistance can be defined as the set of obstacles that arise from health resources standing in the way of seeking or obtaining care. Among these obstacles or deterrent factors are the cost of services, the location of health care sources, and certain characteristics of the ways in which the resources are organized, such as delays in obtaining appointments or in receiving care (34). The Law on the Health System of the Republic of Lithuania stipulates that health care accessibility refers to the conditions recognized by the state that ensure the economic, geographical, and organizational acceptability of health care services for individuals and society (35).

Economic accessibility addresses the financial barriers that may prevent individuals from seeking or receiving care (20), and refers to an individual’s ability to obtain necessary medical services without financial hardship. This concept encompasses the affordability of healthcare services, including costs related to consultations, diagnostics, treatments, medications, and associated non-medical expenses like transportation. Financial barriers can deter individuals from seeking timely care, leading to unmet health needs and adverse health outcomes (36). The indicators of economic accessibility encompass several indicators, such as unmet medical needs due to cost, skipped diagnostics or treatments due to high costs (37), out-of-pocket healthcare expenditures, etc. (38).

Geographical accessibility in healthcare refers to the ease with which individuals can reach healthcare services, considering factors such as physical distance, travel time, and transportation options. It encompasses both spatial accessibility—the distance or travel time to healthcare facilities—and spatial availability, which is the number of healthcare services within a predefined area (39–41).

Organizational accessibility in healthcare refers to how effectively healthcare services are structured to facilitate patient access. This encompasses the alignment of healthcare resources with patients’ needs and preferences, ensuring that services are reachable and usable when required. Key aspects include the availability of appointments, the efficiency of scheduling systems, and the adaptability of healthcare providers to accommodate patients’ varying circumstances (20, 42).

Lithuania is the most rapidly aging country in Europe, presenting growing challenges for healthcare services. Older people have distinct health problems and healthcare needs compared to other age groups. Furthermore, they encounter significantly more difficulties in accessing health services. These challenges are expected to intensify during “abnormal” life situations, such as the COVID-19 pandemic. This study aims to investigate the perceived accessibility of healthcare services among older adults during the COVID-19 pandemic in Lithuania, with a particular focus on geographical, organizational, and financial accessibility, subjective unmet needs and correlations between perceived accessibility and socio-demographic factors, and self-rated health.

## 2 Materials and methods

### 2.1 Research organization

The study was conducted on January 5–17, 2024, in Lithuania. 1,503 Lithuanian residents 65 years of age and older were interviewed by means of an anonymous survey (the response rate 36%; Table 1). Data collection was carried out through face-to-face interviews at respondents’ homes by *Vilmorus Ltd.*, an independent public opinion and market research institution. A team of professional interviewers conducted the interviews, each lasting between 25 and 40 min. The survey encompassed 34 municipalities, including 26 cities and over 50 villages. All participants received information about the study, and participation was both voluntary and anonymous. Verbal consent was obtained from all participants before the interviews.

**Table 1:** Peculiarities of conducting the survey.

Selected contacts	n	%
Participated in the survey	1,503	36.0
Refused to participate	432	10.4
No one was at home	918	22.0
Ineligible due to age/gender	962	23.0
Other (locked stairwell, angry dog, respondent drunk, impossible to communicate, dangerous to enter, uninhabited house, etc.)	360	8.6
Total contacts	4,175	100

### 2.2 Sampling

The survey targeted Lithuanian residents aged 65 and older, employing a multi-stage, probabilistic, and proportional sampling technique. This approach ensured equal representation across various demographic groups and regions of Lithuania. The sample was designed to reflect the demographic distribution of the population by gender and place of residence. Participant selection was based on the Address Register of the Republic of Lithuania, managed by the state-owned enterprise Center of Registers. The “route method” was used for household selection.

The survey was conducted across all 10 administrative regions (counties) of Lithuania, with the number of respondents in each county proportional to its population size, as determined by national statistical data. Within each county, cities, towns and rural localities were selected randomly. Streets and starting points for routes were also chosen at random. To ensure a representative respondent within each household, the “youngest male rule” was applied. If the selected individual was unavailable, a subsequent visit was arranged.

The sample size was calculated using the OpenEpi sample size calculator with the following parameters: population size for each gender and place of residence, anticipated frequency of 50%, a 5% margin of error, and a design effect 1.

### 2.3 Contingent

The distribution and grouping of respondents according to socio-demographic characteristics is presented in Table 2. Lithuanian residents aged 65–96 years participated in the study. Median age was 73 years, mean - 74.04 years (standard deviation 6.37 years). The distribution of the research participants by age, gender and place of residence did not differ significantly from that of the whole population of Lithuania, so the study sample can be considered representative of the population of Lithuania.

**Table 2:** Distribution of respondents according to socio-demographic characteristics and self-rated health.

Factors	Ungrouped responses	n	%	Grouped responses	n	%
Gender	Males	528	35.1	—	—	—
	Females	975	64.9			
Age (years)	65–69	437	29.1	65–74	813	54.1
	70–74	376	25.0	75–84	589	39.8
	75–79	392	26.1	85 and older	101	6.7
	80–84	197	13.1			
	85–96	101	6.7			
Education	Elementary or lower	42	2.8	Lower than secondary	179	11.9
	Main / unfinished Secondary / vocational without secondary	137	9.1	Secondary (secondary + professional with secondary)	628	41.9
	Secondary	332	22.1	Higher and college (higher /technical + college)	415	27.7
	Professional with secondary	296	19.7	University	278	18.6
	Higher (and technical)	388	25.8			
	College	27	1.8			
	University	278	18.5			
	Did not indicate	3	0.2			
Main occupation	Pensioner	1,309	87.1	Pensioner	1,309	87.1
	Disabled	27	1.8	Disabled (disabled + disabled pensioner)	56	3.7
	Working	71	4.7	Working (working +working pensioner)	138	9.2
	Working pensioner	68	4.5			
	Disabled pensioner	29	1.9			
Number of persons in the household	One	617	41.0	One	617	41.0
	Two	746	49.6	Two and more	886	59.0
	Three	96	6.4			
	Four	33	2.2			
	Five	10	0.6			
	Six	2	0.1			
Marital status	Single	48	3.2	Married/living together	735	48.9
	Married	670	44.6	Single (single, divorced, widowed)	768	51.1
	Living together without marriage	65	4.3			
	Divorced	161	10.7			
	Widowed	559	37.2			
Income (after tax for each family member per month)	250 EUR or less	22	1.5	500 EUR or less	618	41.5
	251–500 EUR	596	39.7	501–750 EUR	621	41.7
	501–750 EUR	621	41.3	More than 750 EUR	251	16.8
	751–1,000 EUR	197	13.1			
	More than 1,000 EUR	54	3.6			
	Did not indicate	12	0.8			
Place of residence	Vilnius	254	16.9	City	604	40.2
	Kaunas, Klaipėda, Šiauliai, Panevėžys	350	23.3	Town	399	26.6
	Other towns	399	26.6	Rural area	500	33.2
	Rural area	500	33.2			
Self-rated health	Good	64	4.3	Good / pretty good	270	18.0
	Pretty good	206	13.7	Average	888	59.1
	Average	888	59.1	Pretty bad / bad	344	22.9
	Pretty bad	246	16.4			
	Bad	98	6,5			

### 2.4 Research instrument

An original questionnaire of 74 questions was developed for the study. This article analyzes 14 questions related to perceived accessibility of healthcare services during the COVID-19 pandemic:questions related to the utilization of healthcare services during the COVID-19 pandemic (questions on actual utilization and reasons for non-provision of services); geographical accessibility (questions on whether patients were satisfied with the travel time to their family doctor, specialist doctor, dentist, hospital, and/or rehabilitation service provider); organizational accessibility (questions on difficulties experienced when scheduling a doctor’s consultation and satisfaction with the waiting time from registration to consultation); and financial accessibility, along with unmet needs due to financial reasons (questions regarding unused healthcare services or postponed diagnostics/treatments due to high costs). Nine questions describing respondents’ socio-demographic characteristics and self-rated healthwere also analyzed.

### 2.5 Data analysis

Statistical data analysis was performed using the IBM SPSS Statistics 29 statistical package. Data was weighted by gender and age. To evaluate the linear relationship between quantitative variables, Spearman’s correlation coefficient was calculated. Spearman’s rank correlation is used for measuring and testing association between two continuous or ordered categorical responses. The correlation was considered weak when *r* < 0.3, moderate when 0.3 ≤ *r* < 0.7, and strong when *r* ≥ 0.7. To identify the most vulnerable 65-year-olds and older population groups, research participants’ answers to questionnaire questions were compared according to socio-demographic factors and health characteristics using the Chi-square (χ^2^) criterion, and the z criterion for pairwise comparison of frequencies. Associations were considered statistically significant when the probability of the mistake (*p*) was <0.05.

## 3 Results

### 3.1 Healthcare services utilization during COVID-19 pandemic

**Figure 1:**
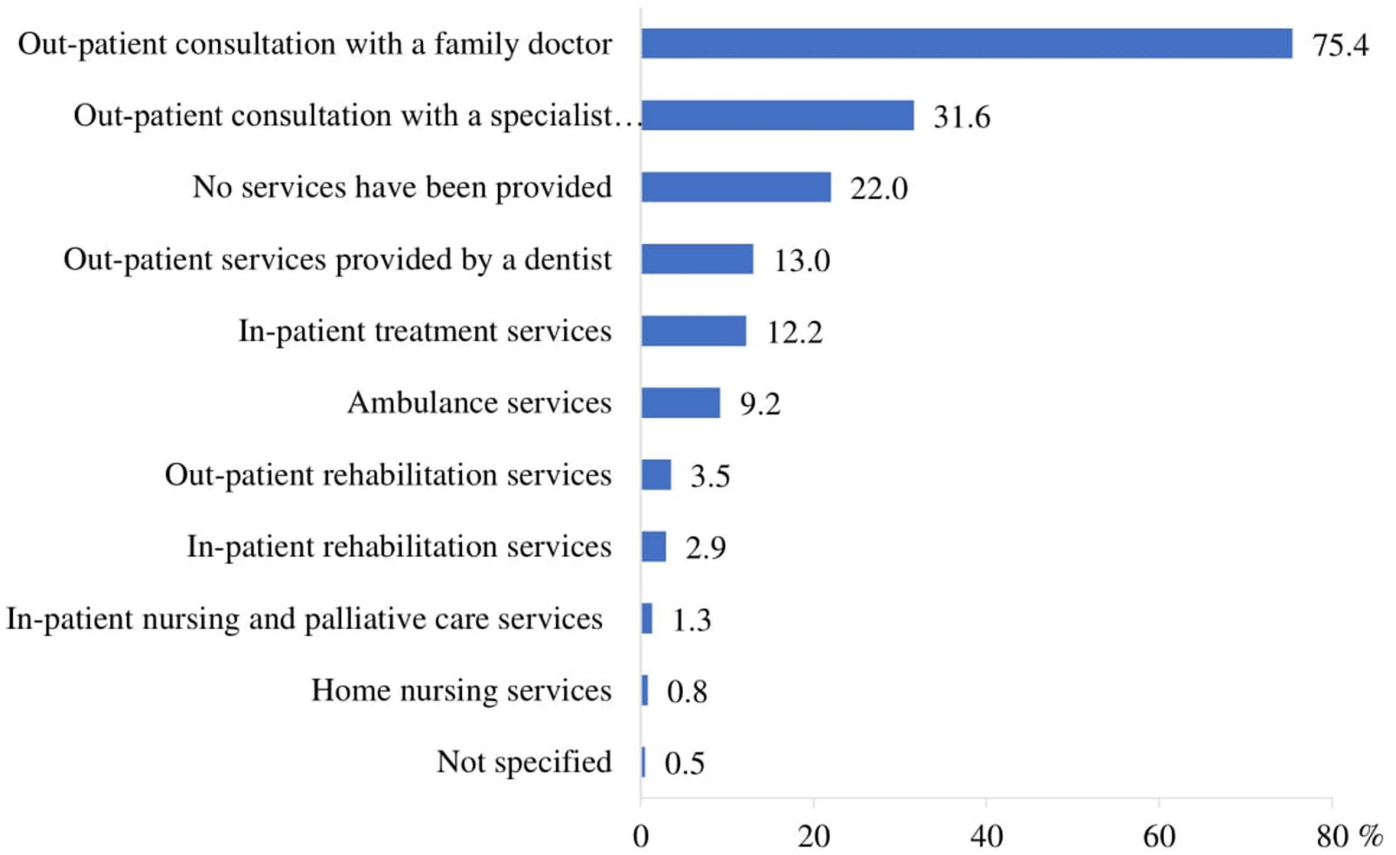
Healthcare services utilization during the COVID-19 pandemic (*n* = 1,503).

The most commonly used healthcare services during the COVID-19 period were out-patient consultations with a general practitioner (75.4%) and a doctor specialist (31.6%). 22.0% of the respondents reported that they did not receive any healthcare services (Figure 1).

**Figure 2:**
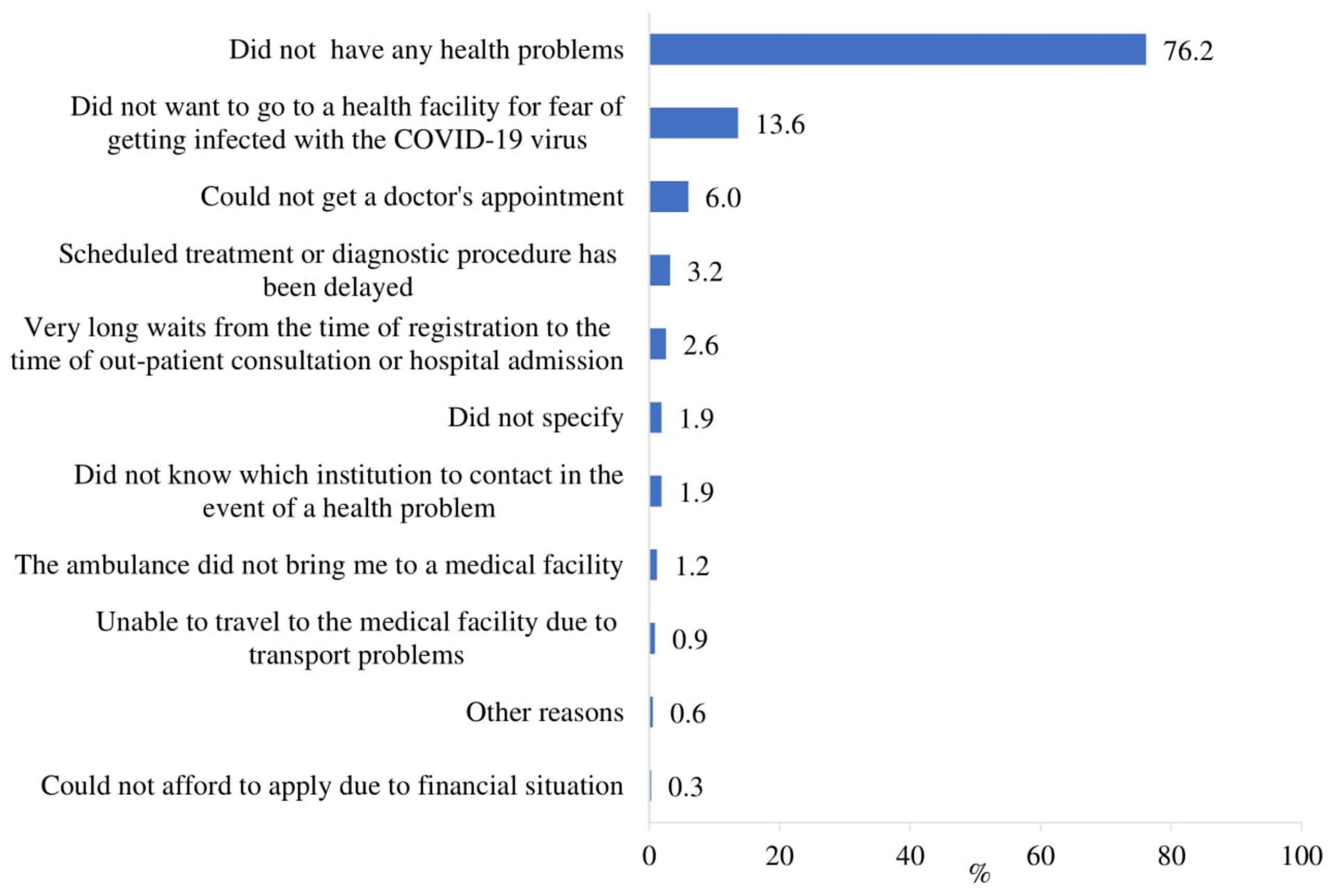
Reasons for not provided healthcare services during the pandemic (*n* = 331).

When asked to indicate the reasons for not receiving healthcare services during the pandemic, most respondents indicated that they did not have any health problems (76.2%), and did not want to go to a health facility because they were afraid of getting infected with the COVID-19 virus (13.6%). 6.0% of respondents could not get a doctor’s appointment, 1.9% did not know which institution to contact in the event of a health problem and 0.3% could not afford to seek care due to their financial situation (Figure 2).

The main reasons for not receiving healthcare services during the COVID-19 pandemic were statistically significantly (*p* < 0.05) related to the respondents’ place of residence and education. Respondents living in cities were more likely than those living in towns or rural areas to report that they had no health problems, those living in rural areas were more likely than those living in urban areas to report that they were afraid to go to a health facility for fear of getting infected with the COVID-19 virus, and those living in towns were more likely than those living in cities to say that they could not get a doctor’s appointment (Supplementary Figure 1). Respondents with lower than secondary education were more likely than those in higher education groups to have not visited a doctor because they were afraid of infection and could not get a doctor’s appointment, and less likely to report good health as a reason for not visiting (Supplementary Figure 2).

### 3.2 Geographical accessibility of healthcare services

**Figure 3:**
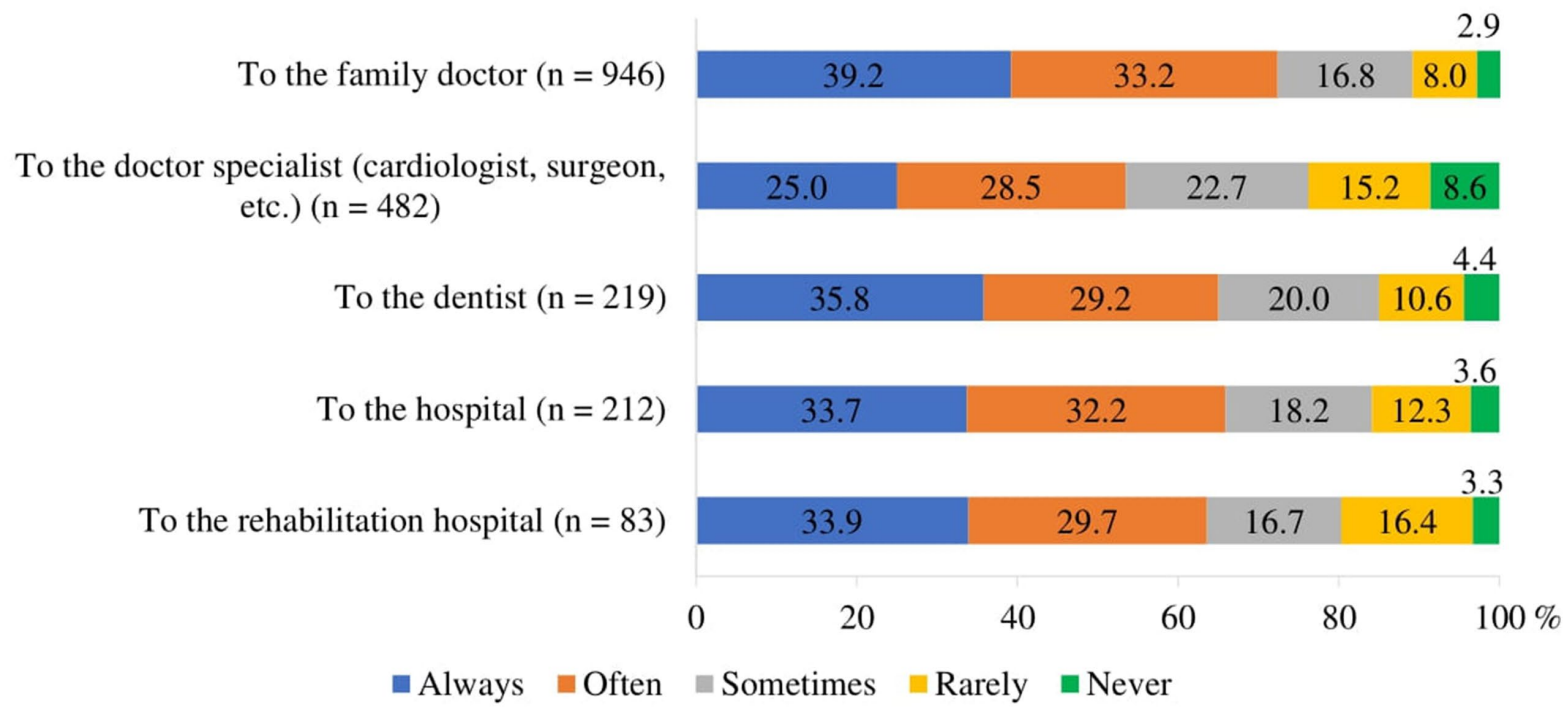
Respondents’ satisfaction with travel time to medical facility during the COVID-19 pandemic (of those who traveled).

To assess geographical accessibility, respondents were asked the question: “During the COVID-19 pandemic, were you satisfied with the travel time to the family doctor, doctor specialist, dentist, hospital and rehabilitation services provider facility?.” The majority of respondents who needed healthcare services stated that they were always or often satisfied with the travel time to the family doctor (71.4%), to the hospital (65.9%), to the dentist (65.0%) and to the rehabilitation hospital (63.6%), while more than half (53.5%) were also satisfied with the travel time to the specialist (Figure 3).

The study found weak statistically significant linear correlations between respondents’ satisfaction with travel time to medical facilities during the COVID-19 pandemic and their education, income, and self-rated health (Table 3):

- the lower the education and the poorer the health, the less satisfied the respondents were with the travel time to the family doctor, specialist and dentist (*p* < 0.001);
- the lower the income, the less satisfying the travel time to the family doctor and dentist (*p* < 0.001).

**Table 3:** Correlations of respondents’ satisfaction with travel time to healthcare facility during the COVID-19 pandemic with socio-demographic and health characteristics (Spearman’s correlation coefficients).

Satisfaction with travel time	Age	Place of residence	Education	Number of persons in the household	Income	Self-rated health
To the family doctor	−0.001	0.024	0.174*	0.004	0.120*	0.121*
To the doctor specialist	0.021	0.026	0.163*	−0.045	0.052	0.131*
To the dentist	0.021	0.055	0.366*	−0.015	0.183*	0.208*
To the hospital	−0.011	0.036	0.061	0.016	−0.046	0.013
To the rehabilitation hospital	−0.020	0.095	0.037	0.006	−0.026	0.018

*p < 0.001.

Respondents were divided into two groups according to their satisfaction with the travel time to the medical facility: satisfied (always or often satisfied) and dissatisfied or partially satisfied (sometimes, very rarely satisfied or never satisfied). Satisfaction with travel time differed statistically significantly (*p* < 0.05) across socio-demographic and self-rated health status groups. Satisfaction with travel time to the family doctor was higher among those living in towns compared to those living in cities or rural areas (Supplementary Figure 3). Those with higher than secondary education were more satisfied with the travel time to the family doctor and doctor specialist compared to lower education groups, while those with higher and lower than secondary education were more satisfied with the travel time to the dentist compared to the other education groups (Supplementary Figure 4) Satisfaction with travel time also depended on the respondents’ self-rated health: the better the self-rated health, the higher the satisfaction with the travel time to the family doctor, specialist and dentist (Supplementary Figure 5).

### 3.3 Organizational accessibility of healthcare services

**Figure 4:**
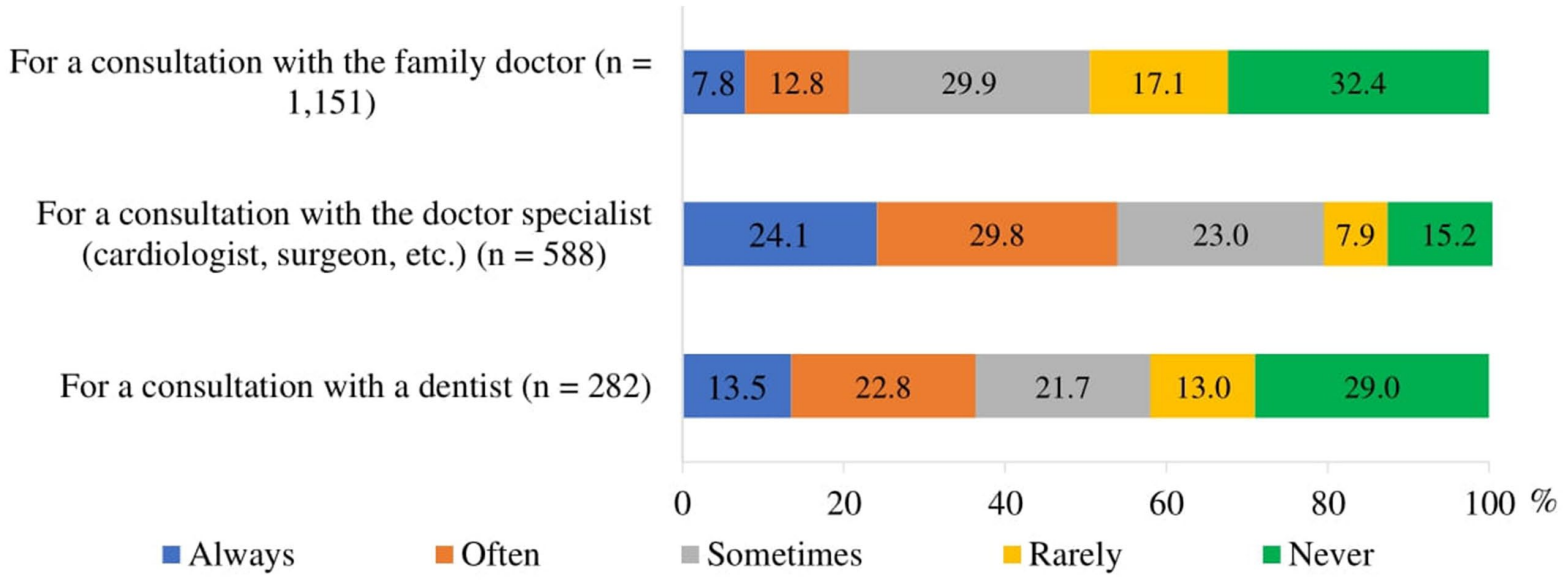
Proportion of respondents who experienced difficulties in getting an appointment for a healthcare consultation during the COVID-19 pandemic (among those who were trying to make an appointment).

To assess organizational accessibility, respondents were asked the question: “During the COVID-19 pandemic, did you experience difficulties when getting an appointment to family doctor’s, doctor specialist’s and dentist consultation?.” Of all respondents who made an appointment for healthcare services, the highest proportion always or often experienced difficulties when making an appointment with a specialist (53.9%), while the lowest proportion had difficulties when making an appointment with a family doctor (20.6%). 36.3% of respondents always or often experienced difficulties when getting an appointment with a dentist (Figure 4).

Weak and statistically significant linear correlations were found between the frequency of difficulties experienced by respondents in getting an appointment for healthcare consultation during the COVID-19 pandemic and their place of residence, education, income, and self-rated health (Table 4):

- residents of larger settlements were more likely than residents of smaller settlements to have experienced difficulties in getting an appointment with a family doctor and less likely to have experienced difficulties in getting an appointment with a specialist (very weak correlation; *p* < 0.05);
- the lower the respondents’ educational level and the poorer self-rated health, the more difficulties they had in getting an appointment with a family doctor, a specialist and a dentist (*p* < 0.001);
- those with lower incomes were more likely to experience difficulties in getting an appointment with a specialist and a dentist (*p* < 0.001).

**Table 4:** Correlations of the frequency of difficulties experienced by respondents in getting an appointment with a doctor during the COVID-19 pandemic with socio-demographic and health characteristics (Spearman’s correlation coefficients).

Difficulties in getting an appointment with	Age	Place of residence	Education	Number of persons in the household	Income	Self-rated health
A family doctor	0.002	0.073*	−0.110**	0.031	−0.003	−0.124**
A doctor specialist	−0.072	−0.090*	−0.220**	−0.032	−0.142**	−0.172**
A dentist	−0.100	−0.024	−0.283**	0.005	−0.172**	−0.160**

*p < 0.05. **p < 0.001.

Respondents were divided into two groups according to the frequency of difficulties experienced in getting an appointment: those who frequently experienced difficulties (always and often) and those who rarely experienced difficulties or did not experience difficulties (sometimes, very rarely, never). The frequency of experiencing difficulties differed statistically significantly (*p* < 0.05) across socio-demographic and self-rated health status groups. Respondents living in cities were more likely to experience difficulties in getting an appointment with a family doctor compared to those living in rural areas. It was more difficult for women, residents of towns and villages, those with secondary or lower education and those with an income of less than EUR 500 to get appointment with a doctor specialist, compared to other socio-demographic groups of respondents. Women, those with higher and college, and secondary education and those with an income of less than EUR 750 were also more likely to experience difficulties in getting an appointment with a dentist (Supplementary Figures 6–9). Respondents who self-rated their health as bad/pretty bad and average were more likely to have difficulties in getting an appointment with doctors, compared with those who self-rated health as good/pretty good (Supplementary Figure 10).

**Figure 5:**
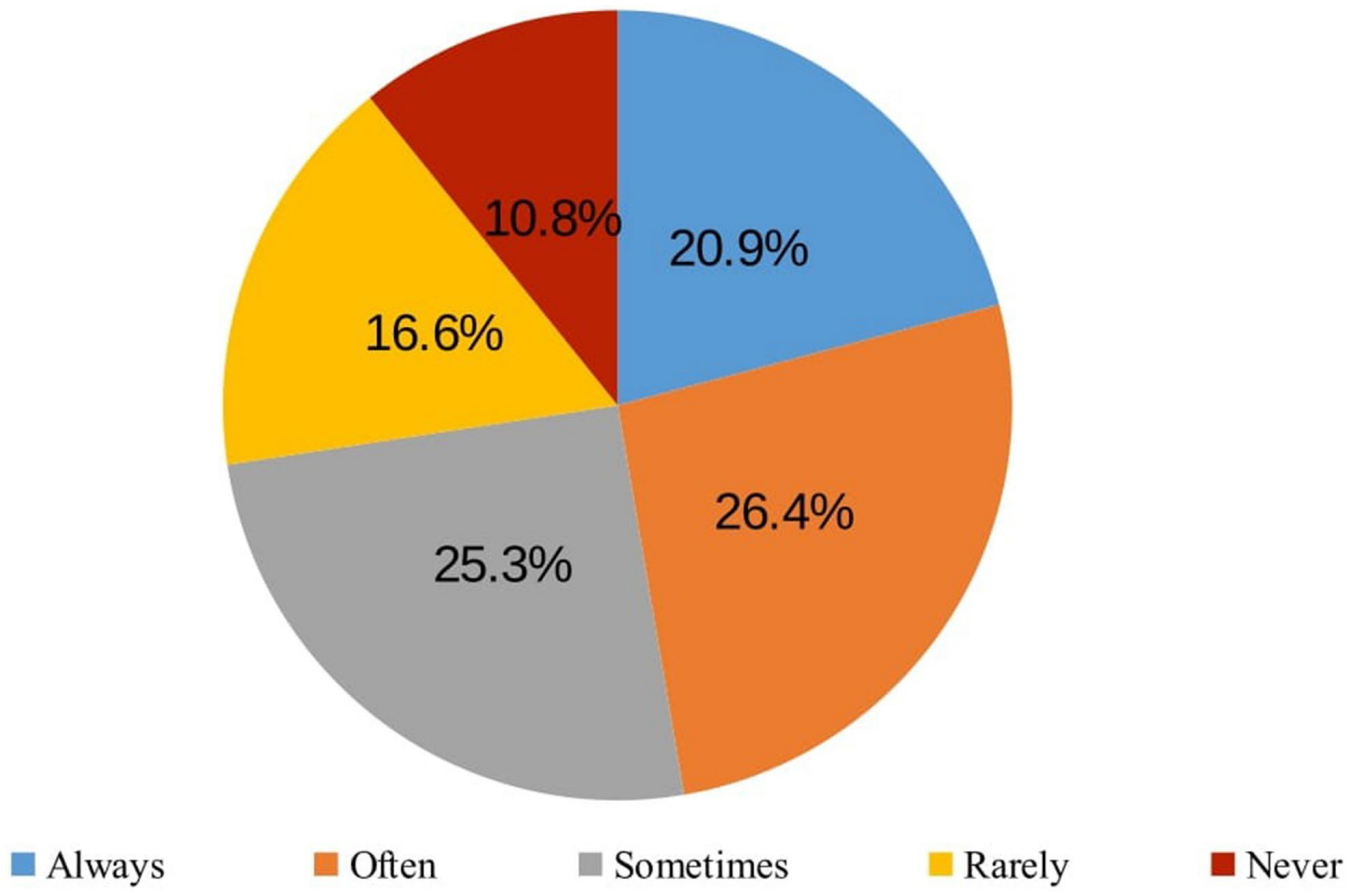
Respondents’ satisfaction with the waiting time from getting an appointment to the doctor’s consultation (regular or remote) during the COVID-19 pandemic (of those who got the appointment, *n* = 1,136).

Additionally, to assess geographical accessibility, respondents were asked the question: “During the COVID-19 pandemic, were you satisfied with the waiting time from getting an appointment to the doctor’s consultation?.” Almost half (47.3%) of the respondents were satisfied with the waiting time from getting an appointment to the doctor’s consultation (Figure 5).

Weak statistically significant linear correlations were found between respondents’ satisfaction with the waiting time from getting the appointment to the doctor’s consultation during the COVID-19 pandemic and their education and self-rated health (Table 5): the lower the education and the poorer the self-rated health, the less satisfied the participants were with the waiting time between getting an appointment and the doctor’s consultation (*p* < 0.001).

**Table 5:** Correlations of respondents’ satisfaction with the waiting time from getting an appointment to the doctor’s consultation during the COVID-19 pandemic with socio-demographic and health characteristics (Spearman’s correlation coefficients).

Satisfaction with the waiting time	Age	Place of residence	Education	Number of persons in the household	Income	Self-rated health
Between getting an appointment and the doctor’s consultation	0.039	0.028	0.109*	−0.040	0.031	0.105*

*p < 0.001.

Respondents were divided into two groups according to their satisfaction with the waiting time from getting an appointment to the doctor’s consultation during the COVID-19 pandemic: satisfied with the waiting time (always and often satisfied) and not satisfied (sometimes, rarely satisfied or not satisfied). Respondents’ satisfaction differed statistically significantly (*p* < 0.05) according to socio-demographic and health status groups. People living in towns and rural areas, those with secondary education and those who self-rated health as bad/pretty bad were less satisfied with the waiting time from an appointment to the doctor’s consultation compared to other socio-demographic groups (Supplementary Figures 11–13).

### 3.4 Financial accessibility of healthcare services

**Figure 6:**
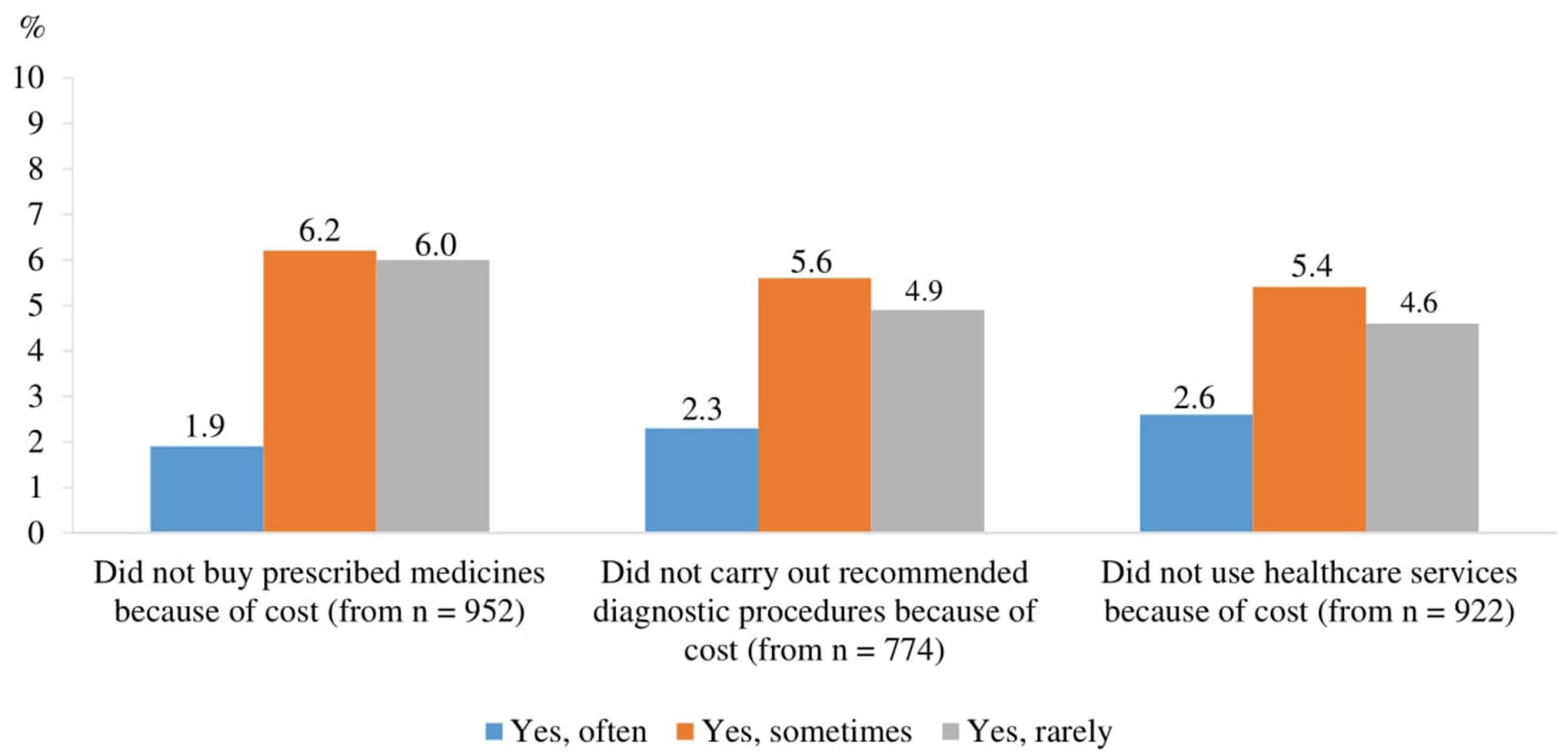
Proportion of respondents whose needs were not met due to financial reasons (the price was too high) during the COVID-19 pandemic (among those who needed services).

To assess financial accessibility, respondents were asked the question: ‘During the COVID-19 pandemic, were there any cases when you did not use healthcare services, did not undergo recommended tests, and/or did not purchase prescribed medications due to financial reasons (too high costs)?.’ A certain proportion of respondents who needed healthcare services during the COVID-19 pandemic reported thatthey did not use them (12.6%), did not carry out recommended diagnostic tests or procedures (12.8%) or did not buy prescribed medicines (14.2%) due to financial reasons (Figure 6).

Weak but statistically significant linear correlations were found between the frequency of respondents’ unmet needs due to financial reasons (too high a price) during the COVID-19 pandemic and their place of residence, income and self-rated health (Table 6):

- the lower the income and the poorer the self-rated health, the more likely they were not to use healthcare services, not to carry out recommended diagnostics/diagnostic procedures and not to buy prescribed medicines due to financial reasons (*p* < 0.001); for the association between income and not carrying recommended diagnostics/diagnostic procedures (*p* < 0.05);
- residents of larger settlements were more likely not to carry out recommended diagnostics/diagnostic procedures due to financial reasons (*p* < 0.05).

**Table 6:** Correlations of the prevalence of unmet respondents’ needs due to financial reasons during the COVID-19 pandemic with socio-demographic and health characteristics (Spearman’s correlation coefficients).

Unmet respondents’ needs due to financial reason	Age	Place of residence	Education	Number of persons in the household	Income	Self-rated health
Did not use healthcare services	0.006	0.060	−0.021	−0.042	−0.141**	−0.111**
Did not carry out recommended diagnostic tests or procedures	0.004	0.091*	0.026	−0.024	−0.092*	−0.119**
Did not buy prescribed medicines	−0.010	0.061	−0.043	−0.019	−0.129**	−0.136**

*p < 0.05. **p < 0.001.

Respondents were divided into two groups according to financial accessibility: those who did not meet their needs for financial reasons (often, sometimes and rarely) and those who had no problems (used, no problems with cost). The proportion of respondents with unmet needs differed statistically significantly (*p* < 0.05) across socio-demographic groups (Supplementary Figures 14–18) and self-rated health status (Supplementary Figure 19). People living in cities, single people, people with disabilities and people with low incomes were more likely not to use healthcare services for financial reasons. Respondents living in cities, and low-income respondents were also more likely to have missed recommended diagnostic tests or procedures. Those who did not purchase the prescribed medicines were most likely to be residents of cities, non-university educated, single, and with a monthly income of EUR 500 or less.

## 4 Discussion

To the best of our knowledge, this is the largest and most comprehensive study investigating perceptions of healthcare access among Lithuanians aged 65 and over during the COVID-19 pandemic, with a particular focus on geographical, organizational, and financial access, as well as the associations between perceived accessibility and socio-demographic factors. A survey of a representative sample of the Lithuanian population aged 65 and over (1,503 respondents) allows us to make evidence-based assumptions about the healthcare needs of this age group and their perceptions of healthcare accessibility. The results of this study could contribute to decision-making processes aimed at enhancing the resilience of the healthcare system in preparation for future epidemics and pandemics.

The findings of this study provide valuable insights into the perceived accessibility of healthcare services among Lithuanians aged 65 and over during the COVID-19 pandemic. The high frequency of consultations with family doctors (75.4%) underscores the crucial role that primary care providers play in managing the health of older adults, especially during public health emergencies. This reliance on family doctors is consistent with other studies highlighting the importance of primary care in maintaining the continuity of health services amid the pandemic (43–46). Additionally, 31.6% of the older adults consulted with specialists, indicating a substantial need for specialized care even during a period characterized by restricted mobility and healthcare service limitations. This finding aligns with the findings of Moynihan et al. (23), who noted that while primary care was largely maintained, specialist consultations experienced a decline globally during the pandemic due to postponed elective procedures and reduced availability of in-person visits.

22.0% of the respondents reported not receiving any healthcare services, including telehealth; of these, 76.2% indicated that they did not have any health problems. One of the most concerning findings is that 13.6% of respondents reported not receiving any healthcare services because they did not want to go to a health facility due to fear of contracting the COVID-19 virus. This statistic highlights a significant barrier to healthcare access rooted in the fear of infection, which has been a widespread concern during the pandemic. This avoidance behavior can be attributed to the heightened perception of risk associated with COVID-19 among older adults, who are at increased risk for severe illness if infected (2, 47). The fear of contracting the virus in healthcare settings has been well-documented and has led to a decrease in healthcare utilization for both emergency and routine care (29, 48). Our findings are consistent with global reports indicating that fear of infection has deterred older adults from seeking necessary medical care (23, 49, 50). Our study showed that 6.0% of respondents reported not receiving any healthcare services because they were unable to get a doctor’s appointment. This data underscores a critical barrier to healthcare access for older adults during a time when medical care is particularly essential. The inability to secure a doctor’s appointment reflects broader systemic issues within healthcare delivery during the pandemic. Similar trends have been observed in other studies (24, 51). The difficulty in obtaining medical appointments may be influenced by the healthcare system’s transition to telehealth services during the pandemic. In Lithuania, doctor appointments were encouraged through online channels, which a certain proportion of older people may have been unable to use properly. While digital technologies have been a crucial tool for maintaining healthcare delivery during the COVID-19 pandemic, they are not always accessible to older adults who may lack digital literacy, access to technology, or familiarity with virtual platforms (52, 53).

Geographical accessibility is a critical component of healthcare access, impacting patients’ ability to obtain timely and necessary medical care. Kelly C et al. investigated whether there is an association between differences in travel time/travel distance to healthcare services and patients’ health outcomes. Their systematic review concluded that 77% of the included studies showed evidence of an association between worse health outcomes and greater travel distance to healthcare facilities. This was evident at all levels of geography—local, interurban, and international (54). Weiss et al. found that 8.9% of the global population cannot reach healthcare within 1 h if they have access to motorized transport, and 43.3% cannot reach a healthcare facility by foot within 1 h (55). In our study, we did not specifically ask how long it takes older people to reach healthcare providers or by what means (e.g., private transport, public transport, or on foot). Instead, we asked whether they were satisfied with the time it took to reach the required healthcare service. This approach provides a subjective measure of geographical accessibility, reflecting the personal experiences and perceptions of the older population regarding travel time to healthcare services. Our study reveals that the majority of respondents who needed healthcare services were consistently or frequently satisfied with the travel time to various healthcare facilities, including family doctors, hospitals, dentists, and rehabilitation hospitals. Specifically, 71.4% of respondents reported being always or often satisfied with the travel time to their family doctor. This high level of satisfaction underscores the importance of local primary care services, which serve as the first point of contact for most healthcare needs and play a crucial role in managing chronic conditions and providing preventive care (56). Convenient access to family doctors is essential for older adults, who often require regular medical consultations. Similarly, 65.9% of respondents were satisfied with the travel time to hospitals, 65.0% with the time to reach a dentist, and 63.6% with the time to reach a rehabilitation hospital. These findings suggest that essential healthcare services are geographically accessible for a significant portion of the older population. This is particularly important during a pandemic when mobility may be restricted, and timely access to medical care is critical (57). However, the satisfaction rate drops to 53.5% for travel time to specialists, indicating that geographical barriers to specialist care remain a concern for nearly half of the respondents. Access to specialist care is vital for diagnosing and managing complex health conditions that primary care providers may not be equipped to handle. Moreover, the COVID-19 pandemic has underscored the need for alternative healthcare delivery methods to mitigate geographical barriers. Telehealth has emerged as a valuable tool for providing remote consultations, reducing the need for travel, and maintaining continuity of care (58). However, as noted above, telehealth is not a panacea and must be complemented by efforts to improve digital literacy and access to technology among older adults to ensure equitable healthcare access (52, 59).

Organizational accessibility refers to the ease with which patients can navigate healthcare systems to receive timely and appropriate care. This aspect of healthcare access is important, particularly for older adults who often have multiple and complex health needs. Our study revealed that the most common problems experienced by respondents were difficulties in getting an appointment with a specialist (53.9%) and a dentist (36.2%). These findings highlight a substantial barrier to accessing specialized healthcare services, which is particularly troubling given the essential role these services play in managing complex health conditions and maintaining oral health. These results are in line with studies in the US and Germany, which also highlight patients’ inability to get an appointment with specialists (24, 60). The difficulties in getting specialist appointments are consistent with broader trends observed during the pandemic, where healthcare systems worldwide faced increased demand and operational disruptions, leading to delays and reduced availability of specialized care (1, 21, 23). As COVID-19 cases started to rise in early 2020 and hospitalization rates increased, health systems began to postpone non-emergency (elective) procedures to keep capacity available for COVID-19 patients, and to avoid elective patients being infected. This has subsequently led to longer waiting lists and waiting times in virtually all countries (61). Not surprisingly, our study found that less than half of the respondents (47.3%) were satisfied with the waiting time between scheduling an appointment and actually getting the healthcare service during COVID-19 pandemic.

Financial accessibility, or the ability to afford healthcare services, is a significant determinant of healthcare utilization and overall health outcomes, particularly for older adults who often live on fixed incomes and face multiple health challenges. Our study reveals that 12.6% of respondents who needed healthcare services did not use them due to financial constraints. Additionally, 12.8% did not undergo recommended tests or diagnostic procedures, and 14.2% did not purchase prescribed medicines because of the high costs. These figures underscore the substantial impact of financial barriers on healthcare access among the older adults during the pandemic. The inability to afford healthcare services and medications can lead to delayed diagnoses, untreated conditions, and ultimately worse health outcomes. This aligns with the findings from previous studies, which have shown that out-of-pocket costs can significantly deter individuals from seeking necessary care and adhering to prescribed treatments (62, 63). Financial barriers to healthcare access are not unique to Lithuania; similar challenges have been documented globally, particularly among vulnerable populations (64, 65).

Our study shows that elder people living in cities, single people, people with disabilities and people with low incomes were more likely not to use healthcare services for financial reasons; elders living in cities, disabled and low-income respondents were also more likely to have missed recommended diagnostic tests or procedures. Those who did not purchase the prescribed medicines were most likely to be residents of cities, non-university educated, single, disabled and with a monthly income of EUR 500 or less (after tax for each family member per month). Rahman AA et al. present similar results in a systematic review and meta-analysis of unmet needs for healthcare and long-term care among older people (37): the common reasons for unmet healthcare needs were cost of treatment, lack of health facilities, lack of/conflicting time, health problem not viewed as serious, and mistrust/fear of provider. A significant variation in pooled prevalence of unmet healthcare needs due to cost was found by gender (male), educational level (primary or less), self-reported health (poor), and economic status of population (poorest). A study in Japan showed that households with older members are more likely to experience catastrophic health expenditure with different financial consequences compared to those with younger members (66).

The COVID-19 pandemic has exacerbated these financial barriers by increasing healthcare costs and reducing household incomes. A study by Arnault L et al. on economic vulnerability and unmet healthcare needs among the population aged 50 and over during the COVID-19 pandemic in Europe substantiates the existence of significant differences in accessing healthcare during the pandemic according to economic vulnerability and of cumulative effects of economic and medical vulnerabilities. The impact of economic vulnerability is notably stronger among those who were in poor health before the outbreak and thus the oldest individuals (67). On the other hand, the COVID-19 pandemic and its negative aspects are not the main reason of the unmet needs of healthcare services for elder people due to financial reasons in Lithuania. An earlier study showed that Lithuania is one of the European countries with the worst relative prevalence of unmet needs for older individuals with healthcare needs (68). Lithuania is a country which provides universal health coverage for the population. Universal health coverage means that all people can access essential health services without incurring financial hardship. Even in countries with good service coverage and financial protection, the progress toward universal health coverage may decelerate or be limited (66). Ensuring financial accessibility to healthcare is essential for improving health outcomes and maintaining the well-being of the older population, especially in times of public health crises.

In general, ensuring adequate healthcare for the population aged 65 and over is important in several respects. Older people have a higher risk of (re)developing chronic diseases. Ensuring access to appropriate healthcare services makes it possible to manage these diseases through regular monitoring of health status and changes in health status, prescribing and/or adjusting appropriate treatment and providing preventive care. Proper management of chronic diseases can improve health, reduce complications and improve the quality of life of older people. Older people often take several (or even more) medications, which can increase the risk of adverse drug reactions and drug interaction errors. Adequate access to healthcare services allows monitoring the effects of prescribed medicines, modifying them and/or their doses, monitoring possible side effects and responding to them in a timely manner. Often, older people require multiple services. Adequate access to health services facilitates the identification, coordination and continuity of service needs and ensures that older people receive all the necessary and integrated health services.

To reduce financial barriers to healthcare access for older individuals living in poverty in Lithuania, several strategies could be implemented: (1) Enhancing public health funding: Lithuania’s healthcare system currently prioritizes hospital-based treatment, with insufficient resources dedicated to public health initiatives. Increasing investment in public health services, particularly those targeting older adults, can improve access to preventive care and reduce long-term healthcare costs (69). (2) Addressing out-of-pocket expenses: High out-of-pocket payments and the limited availability of healthcare professionals outside major cities contribute to inequitable access. Policies aimed at reducing these expenses—such as subsidizing transportation costs and increasing the availability of healthcare workers in rural areas—can significantly enhance access for low-income older adults (70). (3) Strengthening long-term care services: The Organization for Economic Co-operation and Development recommends that Lithuania increase its long-term care workforce by 20% by 2040 to maintain the current caregiver-to-older adults ratio. Expanding and integrating long-term care services can ease the burden on families and ensure older adults receive the necessary care (71). (4) Promoting health literacy and preventive care: Developing and implementing health promotion programs tailored to older adults can empower them to manage their health more effectively. This includes providing accessible information on disease prevention, healthy lifestyles, and available healthcare services (69).

Our findings should be considered in the light of several limitations. First, the survey of the Lithuanian population aged 65 and over was conducted in January 2024, almost 3 years after the first wave of the COVID-19 pandemic. Respondents may not have accurately recalled and evaluated their experiences during the COVID-19 pandemic. Second, it is clear that only respondents who successfully survived the COVID-19 pandemic participated in this survey. Third, only respondents who were at home at the time of the survey were interviewed. It is likely that some of the population aged 65 and over may have been in hospital or in a nursing home at the time of the survey, and therefore may have a different perspective on the accessibility of services. All of these reasons may lead to some bias in the responses, but we are confident that the survey of the Lithuanian population aged 65 and over represents a reliable perceptions of the healthcare access during the COVID-19 pandemic.

## 5 Conclusion

This study highlights significant patterns and barriers in healthcare services utilization among Lithuanian residents aged 65 and older during the COVID-19 pandemic, emphasizing both accessibility challenges and socio-demographic disparities.

Healthcare Services Utilization. The study found that the most frequent consultations during the COVID-19 period were with a family doctor (75.4%) and a specialist (31.6%). The majority of respondents accessed general practitioner consultations, while a substantial proportion (22%) reported not receiving any healthcare services. The primary reasons for non-utilization included perceived good health (76.2%) and fear of COVID-19 infection (13.6%), with variations across residence and education levels. This finding underscores the role of perceived risk and public health messaging in influencing healthcare-seeking behavior during health crises.

Geographical Accessibility. Most respondents expressed satisfaction with travel time to healthcare facilities, but satisfaction was significantly associated with socio-economic factors. Those with lower education, lower income, and poorer self-rated health reported less satisfaction, revealing geographical disparities in access. Residents of towns were more satisfied with travel time to family doctors than those in rural areas or cities, highlighting the need for improved transportation infrastructure or healthcare availability in under-served regions.

Organizational Accessibility. A majority of respondents experienced difficulty scheduling appointments with specialists, with lower difficulty levels for family doctor appointments. The most common organizational access problems experienced by respondents were difficulties in getting an appointment with a specialist (53.9%) and a dentist (36.2%). Difficulty in accessing care was more pronounced among respondents with lower education, lower income, poorer self-rated health, and women. These findings indicate systemic barriers in healthcare management that disproportionately affect vulnerable populations, pointing to the need for more efficient appointment systems and patient support services.

Satisfaction with Waiting Times. Less than half of the respondents were satisfied with the waiting time between appointments and consultations. Satisfaction was significantly lower among those with lower education, poorer self-rated health, and rural or town residents. This highlights the impact of health system capacity and socio-economic inequalities on patient experiences during the pandemic, emphasizing the need for policy interventions to reduce waiting times and enhance service efficiency.

Financial Accessibility. Financial barriers prevented a notable proportion of respondents from using healthcare services, undergoing recommended diagnostic tests, or purchasing prescribed medicines. 12.6% of respondents who needed healthcare during the COVID-19 pandemic did not use healthcare services, 12.8% did not carry out the recommended tests or diagnostic procedures, and 14.2% did not buy the prescribed medicines due to financial reasons (too high cost). The burden was greatest among city dwellers, low-income groups, and individuals with disabilities or poorer self-rated health. These results reflect the critical importance of financial support mechanisms and equitable healthcare policies to reduce the economic burden of healthcare on at-risk populations.

## Data Availability

The original contributions presented in the study are included in the article/Supplementary material, further inquiries can be directed to the corresponding author.
