# Supplementary material for: Perceptions of healthcare access among Lithuanians aged 65 and over during the COVID-19 pandemic
Source: Front Public Health. 2025 Mar 4;13:1504049. doi: 10.3389/fpubh.2025.1504049 (PMC11913820; doi:10.3389/fpubh.2025.1504049)
Supplement: Supplementary file 1 [file Data_Sheet_1.PDF]

Supplementary Material

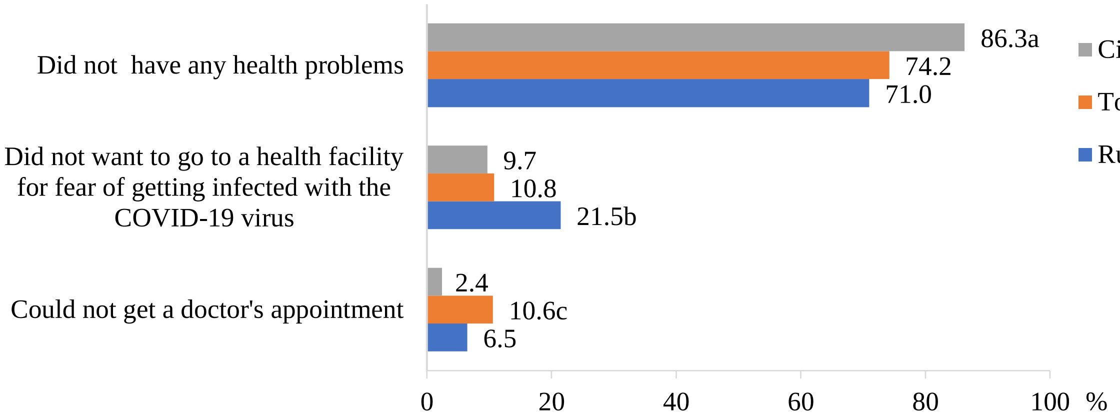

a -p<0.05 compared with town and rural area residents
b- p<0.05 compared with city and town residents

Supplementary Figure 1. Reasons for not receiving healthcare services during the COVID-19 pandemic by place of residence

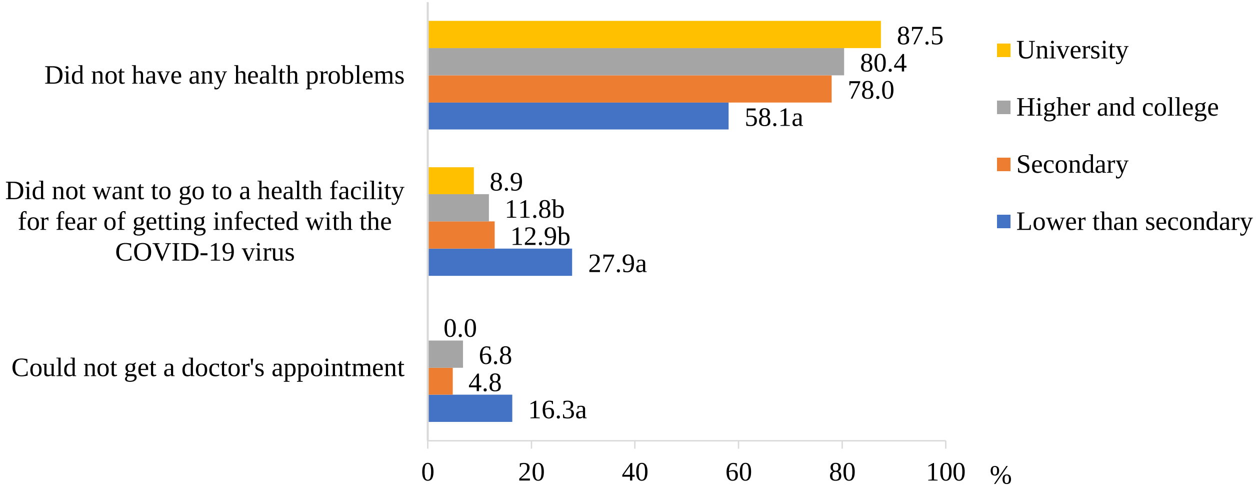

a- p<0.05 compared with all other education
b- p<0.05 compared with university education

Supplementary Figure 2. Reasons for not receiving healthcare services during the COVID-19 pandemic by education

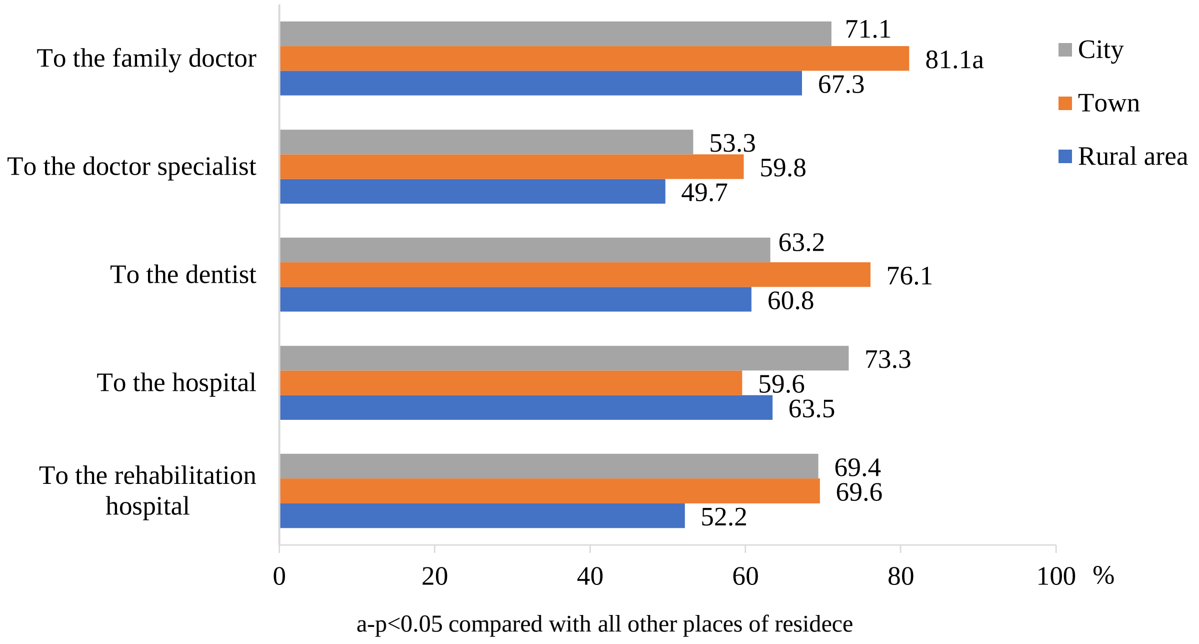

Supplementary Figure 3. Proportion of respondents satisfied with their travel time to a medical facility during the COVID-19 pandemic, by place of residence

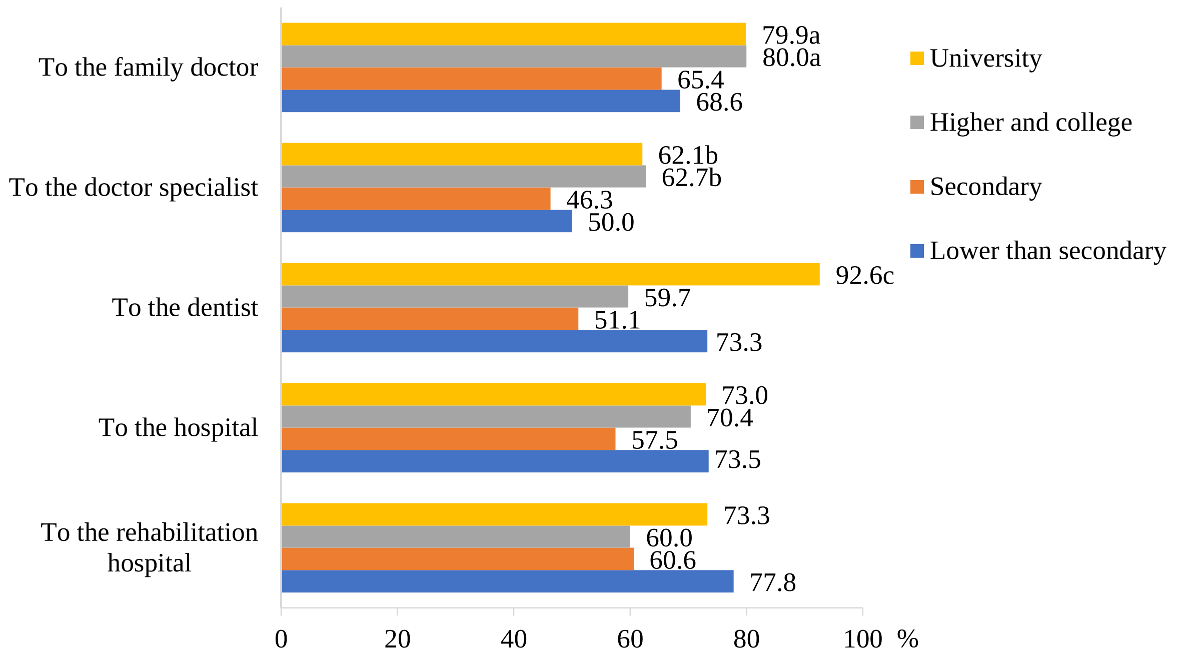

a-p<0.05 compared with secondary and lower than secondary education
b-p<0.05 compared with secondary education
c-p<0.05 compared with all other education

Supplementary Figure 4. Proportion of respondents satisfied with their travel time to a medical facility during the COVID-19 pandemic, by education

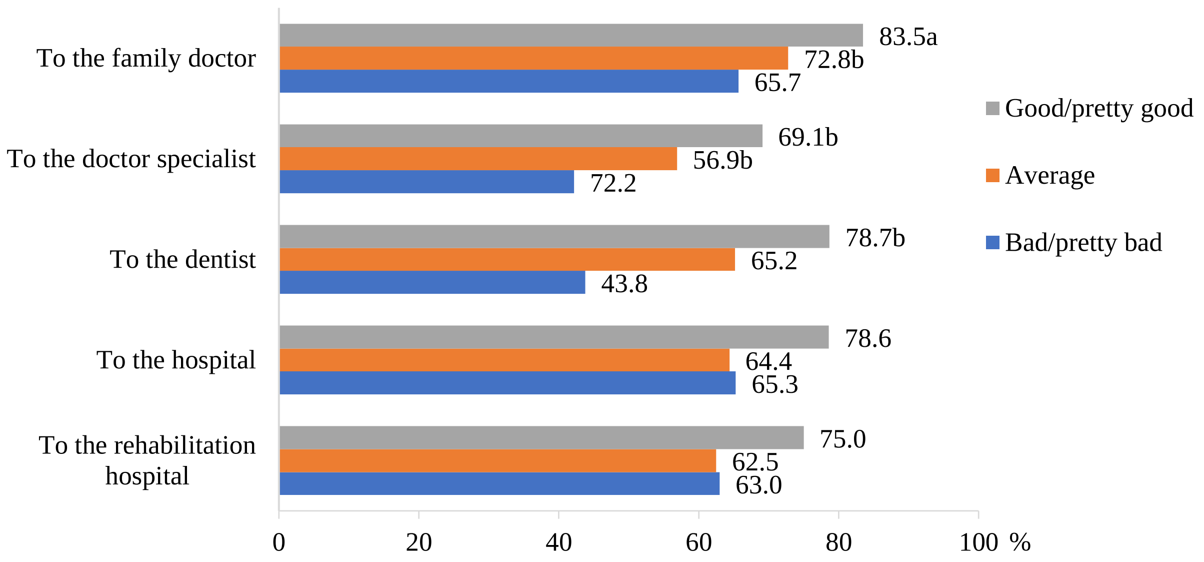

a-- p<0.05 compared with average and bad/pretty bad self-rated health
b- p<0.05 compared with bad/pretty bad self-rated health

Supplementary Figure 5. Proportion of respondents satisfied with their travel time to a medical facility during the COVID-19 pandemic, by self-rated health

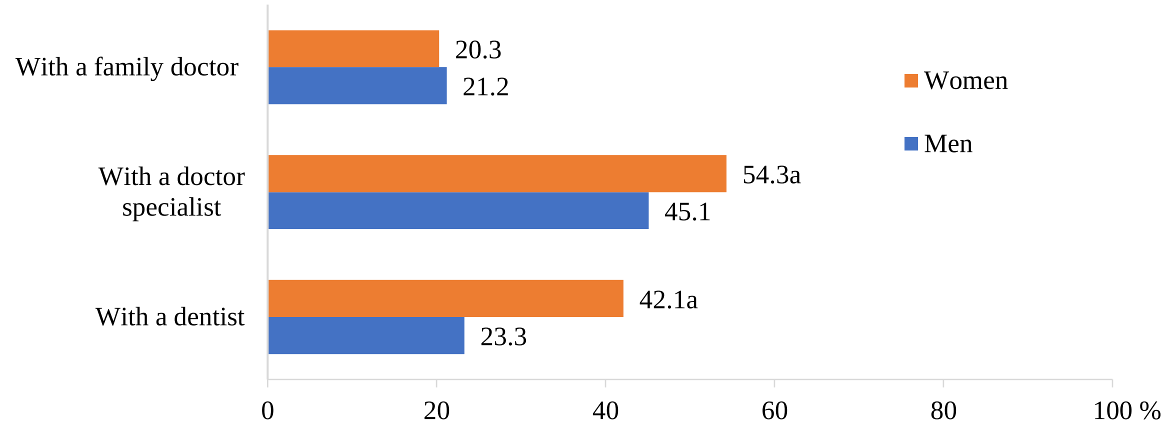

a-p<0.05 compared with men

Supplementary Figure 6. Proportion of respondents who frequently experienced difficulties in getting an appointment with a doctor during the COVID-19 pandemic, by gender

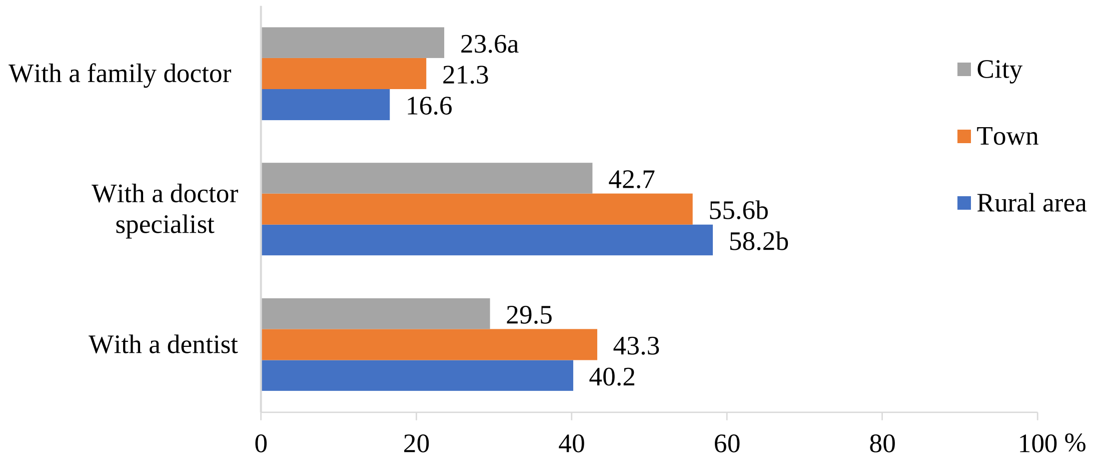

a-p<0.05 compared with rural area residents
b-p<0.05 compared with city residents

Supplementary Figure 7. Proportion of respondents who frequently experienced difficulties in getting an appointment with a doctor during the COVID-19 pandemic, by place of residence

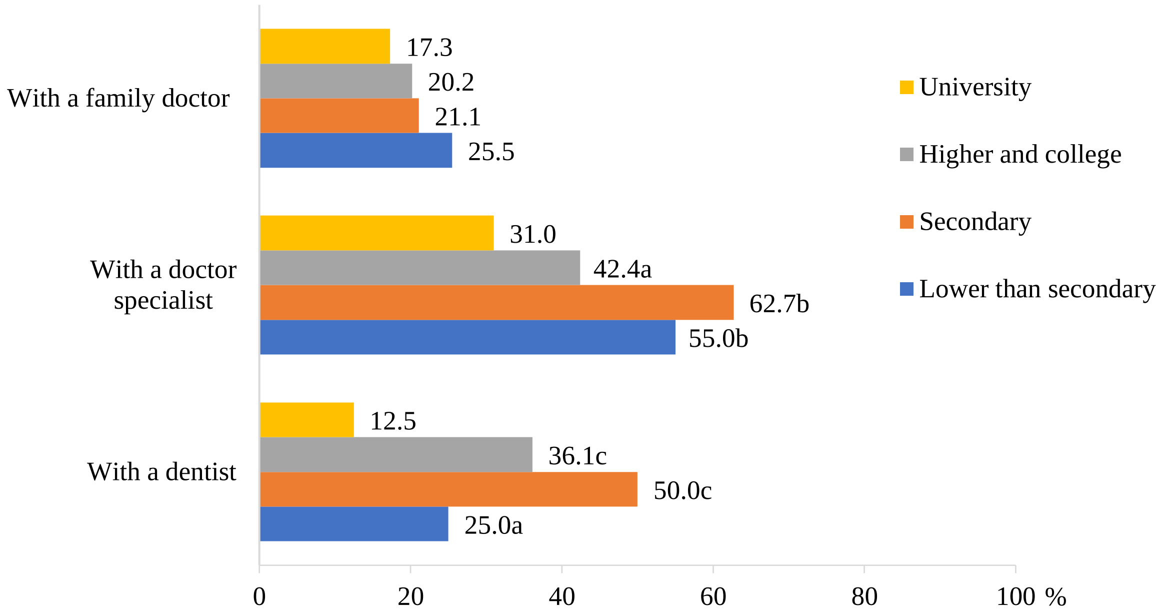

a-p<0.05 compared with university education
b-p<0.05 compared with university and higher and college education
c-p<0.05 compared with all other education

Supplementary Figure 8. Proportion of respondents who frequently experienced difficulties in getting an appointment with a doctor during the COVID-19 pandemic, by education

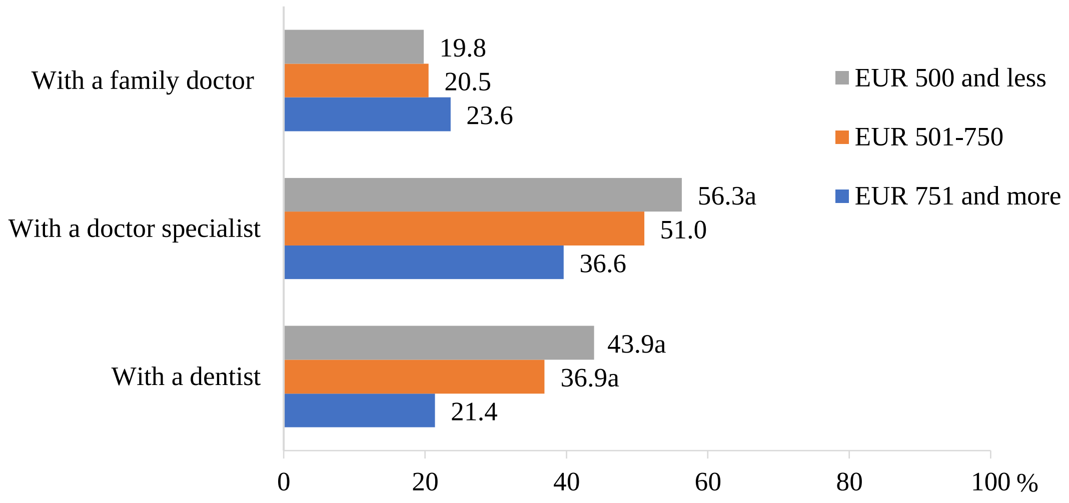

a-p<0.05 compared with EUR 751 and more income

Supplementary Figure 9. Proportion of respondents who frequently experienced difficulties in getting an appointment with a doctor during the COVID-19 pandemic, by income

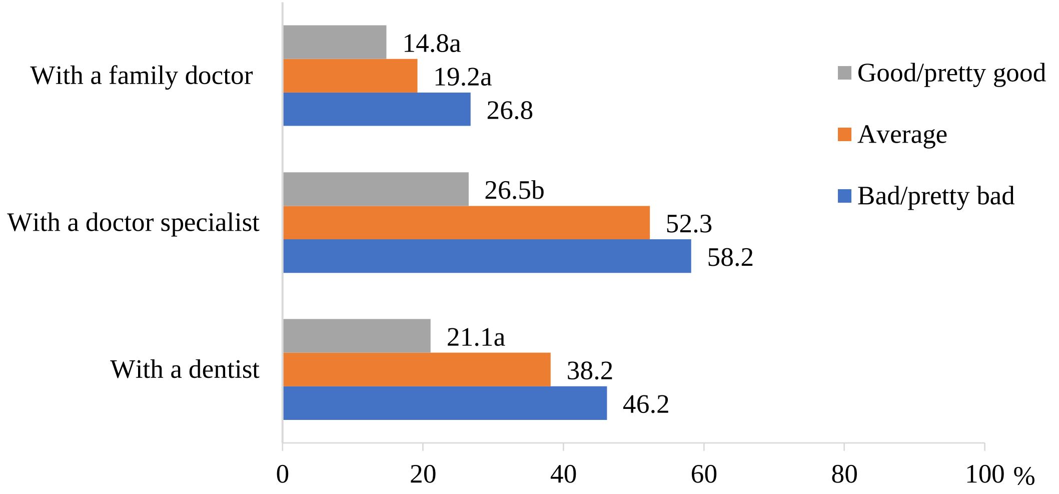

a-p<0.05 compared with bad/pretty bad self-rated health
b-p<0.05 compared with average and bad/pretty bad self-rated health

Supplementary Figure 10. Proportion of respondents who frequently experienced difficulties in getting an appointment with a doctor during the COVID-19 pandemic, by self-rated health

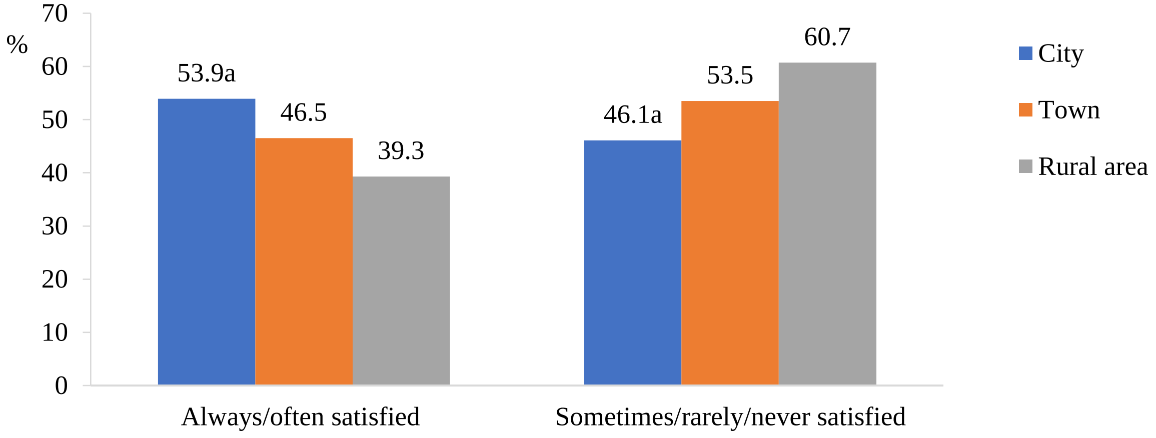

$\chi^2 = 17{,}196;\ df = 2;\ p<0.001$

a- p<0.05 compared with town and rural area residents

Supplementary Figure 11. Respondents' satisfaction with the waiting time from getting an appointment to the doctor's consultation during the COVID-19 pandemic, by place of residence

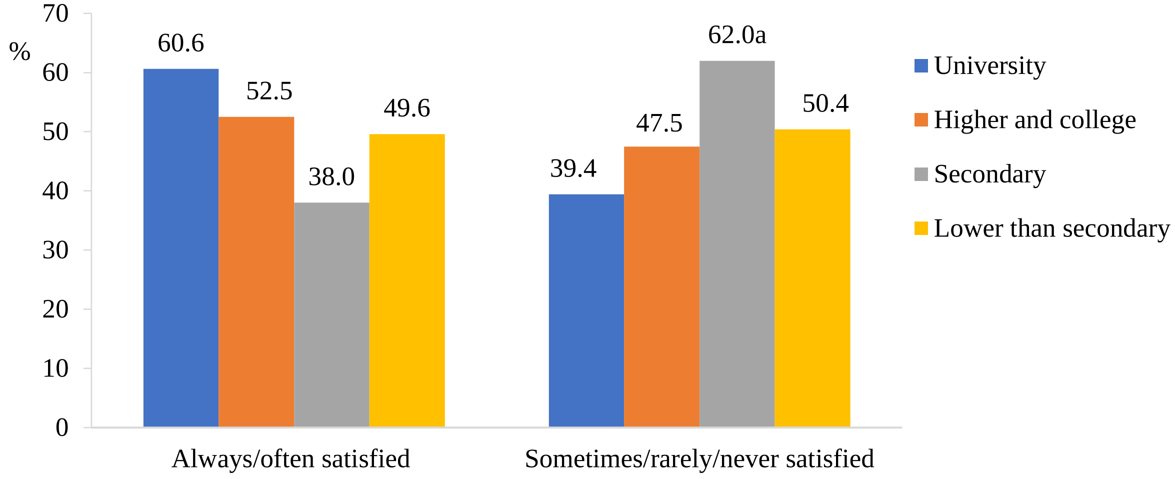

$\chi^2 = 35{,}079; df = 3; p<0.001$

a-p<0.05 compared with other education

Supplementary Figure 12. Respondents' satisfaction with the waiting time from getting an appointment to the doctor's consultation during the COVID-19 pandemic, by education

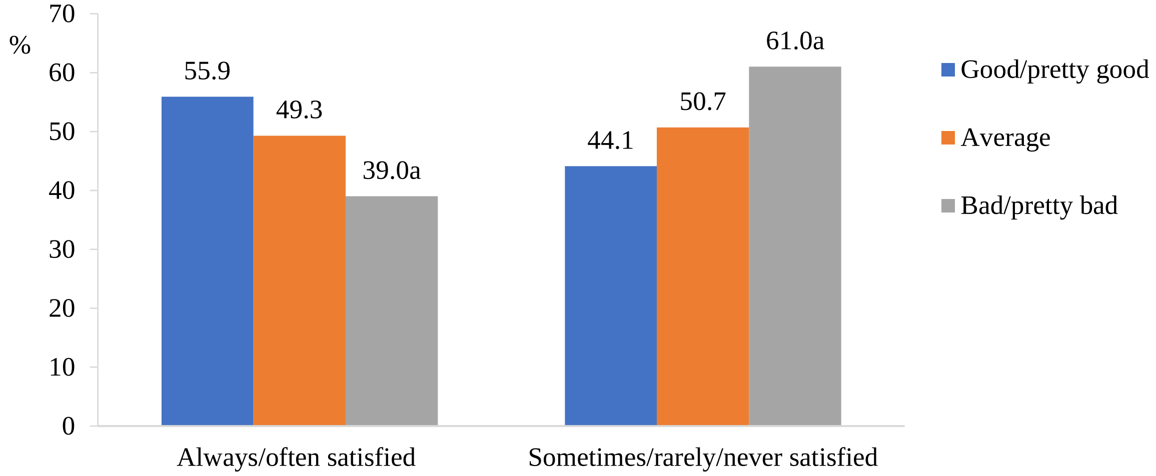

$\chi^2 = 13{,}947; df = 2; p<0.001$

a-p<0.05 compared with good/pretty good and average self-rated health

Supplementary Figure 13. Respondents' satisfaction with the waiting time from getting an appointment to the doctor's consultation during the COVID-19 pandemic, by self-rated health

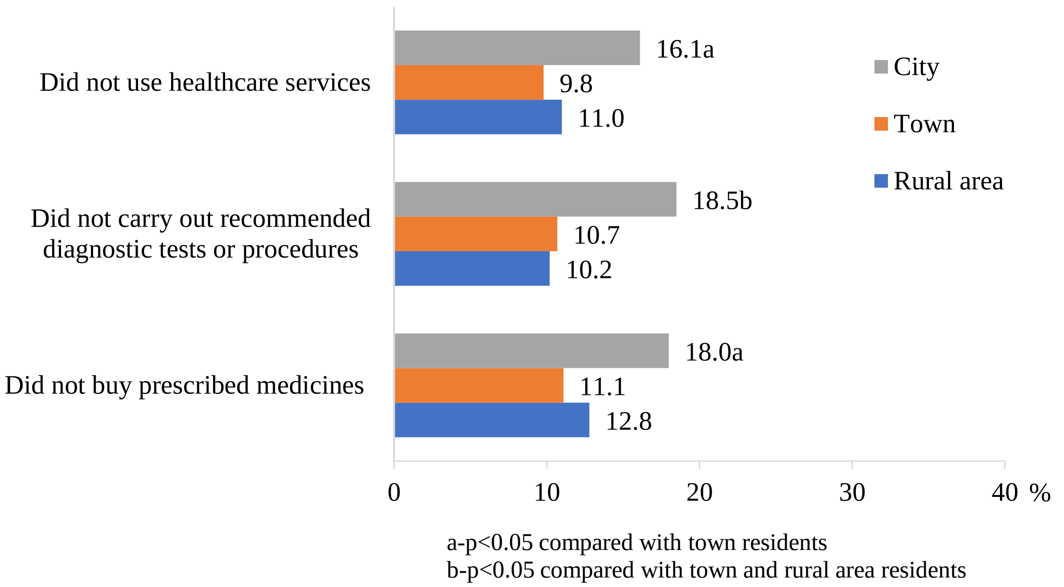

Supplementary Figure 14. Proportion of respondents with unmet (often/sometimes/rarely) healthcare needs due to financial reasons during the COVID-19 pandemic, by place of residence

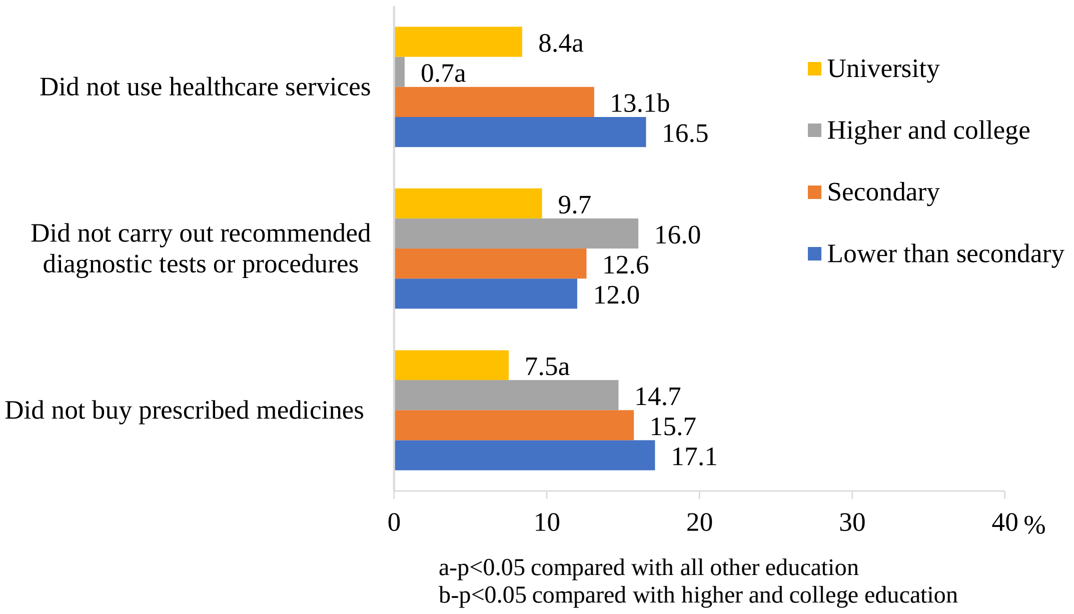

Supplementary Figure 15. Proportion of respondents with unmet (often/sometimes/rarely) healthcare needs due to financial reasons during the COVID-19 pandemic, by education

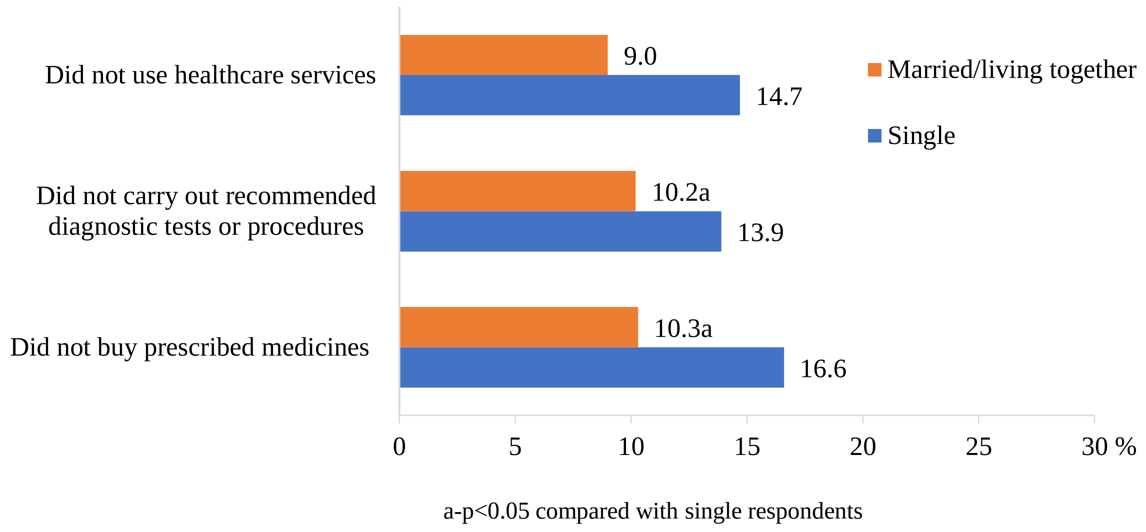

Supplementary Figure 16. Proportion of respondents with unmet (often/sometimes/rarely) healthcare needs due to financial reasons during the COVID-19 pandemic, by marital status

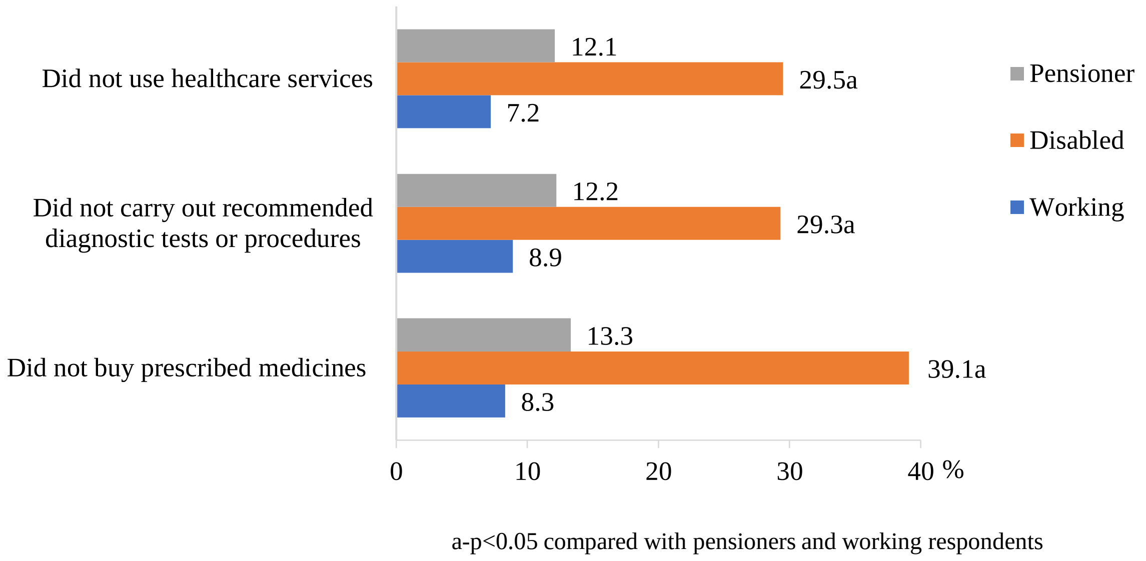

Supplementary Figure 17. Proportion of respondents with unmet (often/sometimes/rarely) healthcare needs due to financial reasons during the COVID-19 pandemic, by main occupation

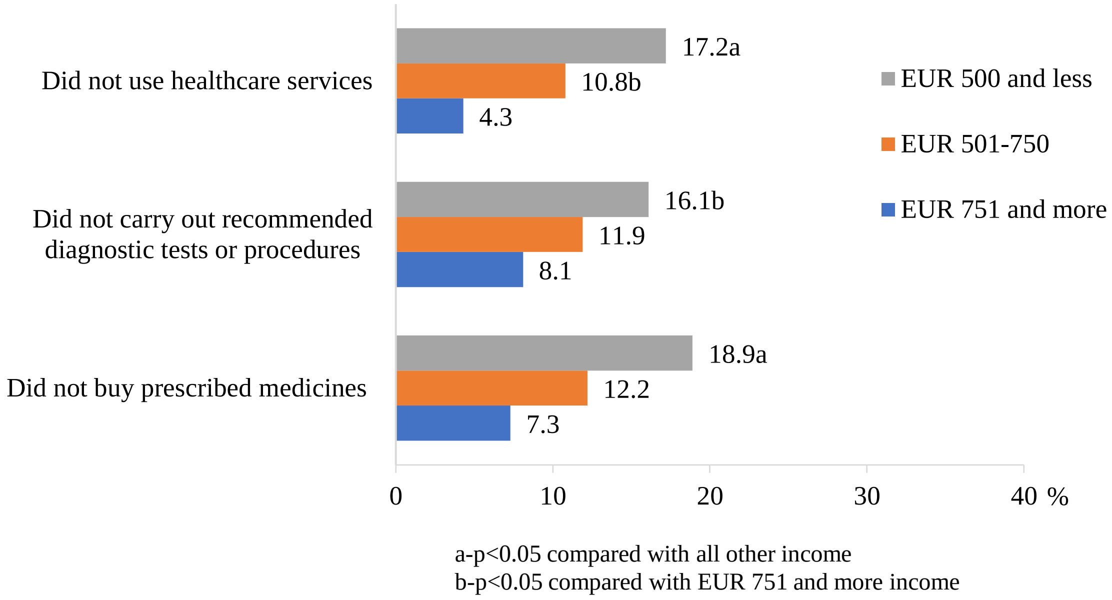

Supplementary Figure 18. Proportion of respondents with unmet (often/sometimes/rarely) healthcare needs due to financial reasons during the COVID-19 pandemic, by income

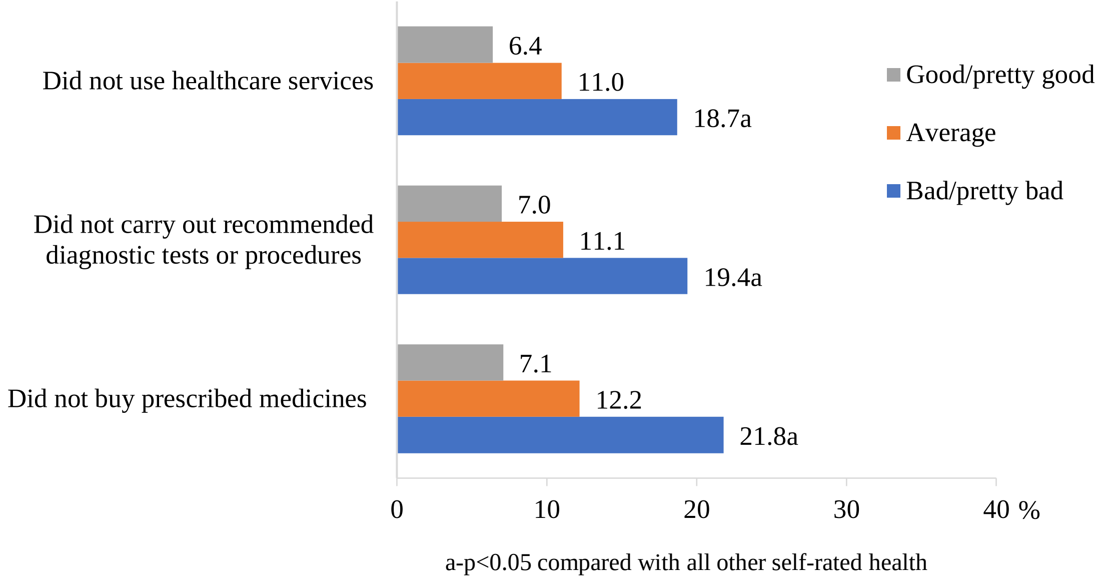

Supplementary Figure 19. Proportion of respondents with unmet (often/sometimes/rarely) healthcare needs due to financial reasons during the COVID-19 pandemic, by self-rated health
